# The Proliferation Index of Erythroid Cells Predicts the Development of Transfusion-dependence in Myelodysplastic Syndrome Patients With Mildly Reduced Hemoglobin Levels at Initial Diagnosis

**DOI:** 10.1097/HS9.0000000000000804

**Published:** 2022-11-09

**Authors:** Stefan G. C. Mestrum, Norbert C. J. de Wit, Eline M. P. Cremers, Roosmarie J. M. Drent, Frans C. S. Ramaekers, Anton H. N. Hopman, Math P. G. Leers

**Affiliations:** 1 Department of Molecular Cell Biology, GROW-School for Oncology and Reproduction, Maastricht University Medical Center, The Netherlands; 2 Department of Clinical Chemistry & Hematology, Zuyderland Medical Center, Sittard-Geleen, The Netherlands; 3 Central Diagnostic Laboratory (CDL), Maastricht University Medical Center, The Netherlands; 4 Department of Hematology, Radboud University Medical Center, Nijmegen, The Netherlands; 5 Nordic-MUbio, Susteren, The Netherlands

Transfusion-dependence in myelodysplastic syndrome (MDS) patients significantly impairs their quality of life and prognosis of survival. Therefore, proper management of this severe complication is of utmost importance.^1^ Current transfusion schedules for MDS patients include frequent visits to hospitals for blood sample collection to determine hemoglobin (Hb) levels and blood transfusion if necessary.^2^ Despite these burdensome situations for the patient, more optimal approaches for management of transfusion-dependence are still under investigation.^3^ In current clinical practice, a very low Hb level at diagnosis is indicative for future transfusion-dependence in MDS patients and is used as a surrogate marker for this severe complication within the revised international prognostic scoring system (IPSS-R).^4^ However, part of the MDS patients with only mildly reduced Hb levels at diagnosis are still at risk to develop transfusion-dependence.^5^ Biomarkers that predict transfusion-dependence in these patients may contribute to more adequate management thereof and may complement the IPSS-R.

In our previous study, the Ki-67 proliferation index of nucleated erythroid cells was shown to be significantly reduced in bone marrow (BM) aspirates from MDS patients (40%) compared to nonmalignant BM (70%), while the interpatient variability in MDS was high.^6^ These findings indicate that this parameter may be an important factor in the development of transfusion-dependence. Therefore, we determined whether the Ki-67 proliferation index of nucleated erythroid cells in the BM at diagnosis (1) is significantly different in MDS patients that developed transfusion-dependence within 1 year after diagnosis compared to patients that did not and (2) is predictive for transfusion-dependence in MDS patients with mildly reduced Hb levels.

In this prospective study, 45 MDS patients were included that underwent BM aspiration for routine diagnostic purposes in the Zuyderland Medical Center (Suppl. Table S1). All patients were diagnosed according to the WHO classification, and the IPSS-R classification was available for 36 of 45 MDS cases. During the follow-up period of 1 year postdiagnosis, 23 patients developed transfusion-dependence as assessed by the International Working Group 2006 response criteria for MDS.^7^ Data were acquired after informed consent in accordance with the Declaration of Helsinki, while also approval for this study was obtained from the Medical Ethical Committee of the Zuyderland Medical Center and Hogeschool Zuyd (METC Z registration nr. 15-N-201). Patients that were being treated with radiotherapy and/or chemotherapy were excluded from analysis. Hb levels in the peripheral blood at diagnosis were determined with the Sysmex XN-9000 hematology analyzer (Sysmex, Kobe, Japan). Ten-color flow cytometry with 7 different antibody panels for immunophenotypic analysis and gating of the total populations of nucleated erythroid cells was performed as described previously.^8^ Gating of the Ki-67 proliferation index of the nucleated erythroid cells in the BM was performed with the Infinicyt v2.0 software package (Cytognos SL, Salamanca, Spain) according to the gating strategy described in our previous publication.^9^ Statistical analysis was performed using the GraphPad Prism 8.0 software package (GraphPad Software, San Diego, USA) and SPSS 26.0 (IBM Corporation, New York, USA). Normality was tested using the Shapiro–Wilk test. The independent T-test and Mann–Whitney U test were used for estimation of differences in Ki-67 proliferation index of nucleated erythroid cells between MDS patients that developed transfusion-dependence and those of patients that remained transfusion-independent during the follow-up period of 1 year. Based on the median Ki-67 proliferation index of nucleated erythroid cells (28%) and median Hb levels (9.3 g/dL), the individual MDS patients were stratified in very low and mildly reduced groups for each of the 2 parameters. Linear regression in combination with the Spearman rho coefficient was used to assess the correlation between the Ki-67 proliferation index of nucleated erythroid cells in the BM and Hb levels in the peripheral blood, both at initial diagnosis. Kaplan–Meier curve analyses, univariable and multivariable Cox-regression were then used to determine whether the Ki-67 proliferation index of nucleated erythroid cells in the BM and Hb levels in the peripheral blood at initial diagnosis were associated with the development of transfusion-dependence of MDS patients during the follow-up period of 1 year postdiagnosis. Following 3 different procedures (entering of all parameters at once, forward and backward elimination), age, the Ki-67 proliferation index of nucleated erythroid cells and Hb levels at initial diagnosis were included in the Cox-regression model.

When comparing the Ki-67 proliferation index of the nucleated erythroid cell population in the BM between the group of transfusion-dependent and that of transfusion-independent MDS patients (Figure 1A), a decreased Ki-67 proliferation index was seen in MDS patients that developed transfusion-dependence as compared to those that did not (*P* = 0.027). A positive trend was seen between the Ki-67 proliferation index of nucleated erythroid cells and Hb levels, although this did not reach statistical significance (*P* = 0.123; Figure 1B). Strikingly, 5 of 8 (63%) MDS patients that showed only mildly reduced Hb levels became transfusion-dependent within 1 year postdiagnosis, while a very low Ki-67 proliferation index as found in these patients. Furthermore, 10 of 28 (36%) patients with very low Hb levels remained transfusion-independent. Most of these patients displayed a mildly reduced Ki-67 proliferation index. Almost all Ki-67 very low/Hb very low patients developed transfusion-dependence and All patients with mildly reduced Ki-67 and Hb levels remained transfusion-independent patients.

**Figure 1.**
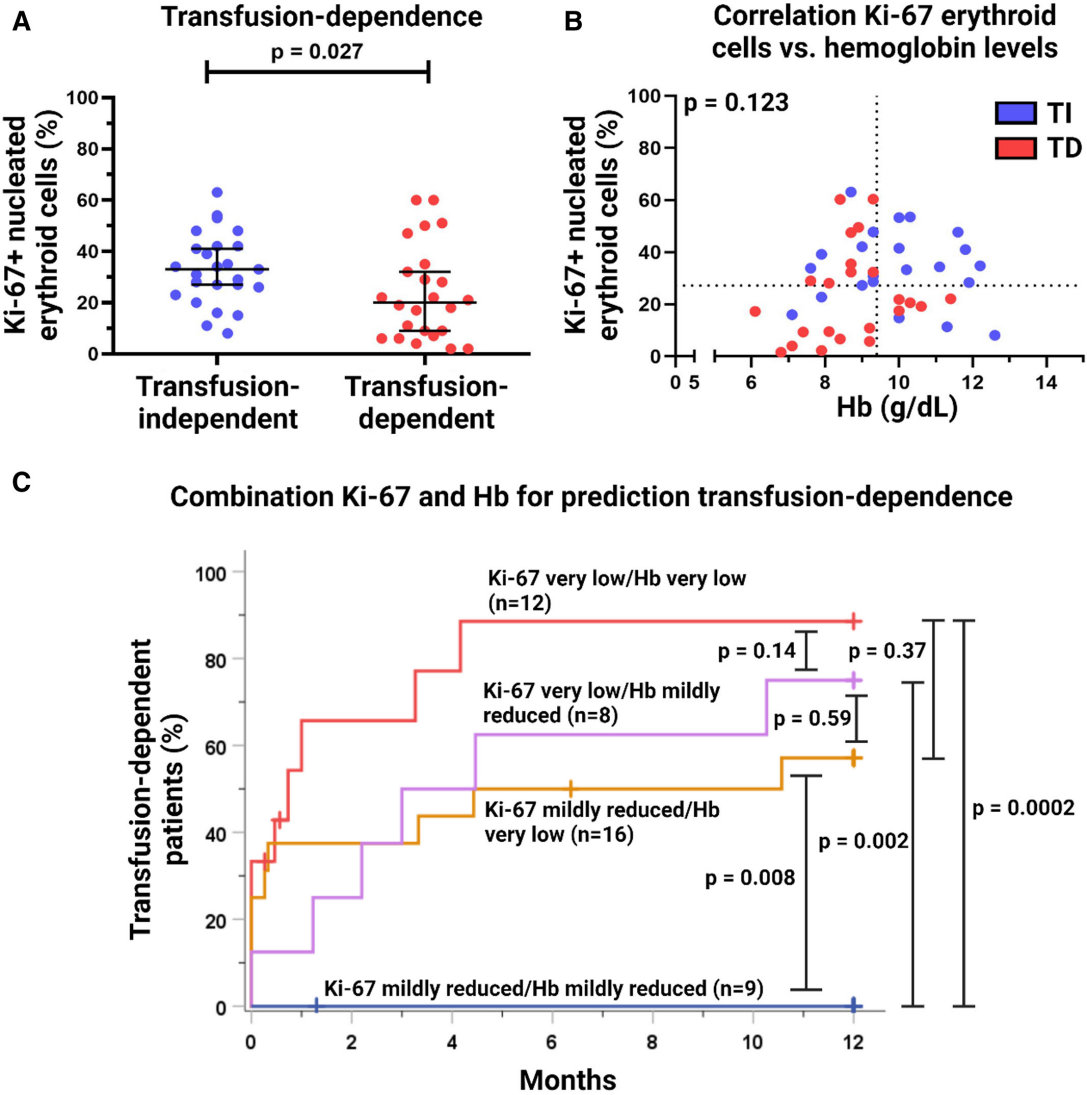
**The relationship between the Ki-67 proliferation index, Hb levels and the development of transfusion-dependence of MDS patients.** (A) Graphic representation of the Ki-67 proliferation index of the total population of nucleated erythroid cells in MDS patients that developed transfusion-dependence as compared to those that did not. Horizontal lines depict the median Ki-67 proliferation index, and the whiskers depict the 95% confidence interval. The average Ki-67 proliferation index of nucleated erythroid cells was significantly decreased in MDS patients that developed transfusion-dependence within 1 year postdiagnosis as compared to the patients that remained transfusion-independent. (B) Assessment of the degree of correlation between the Ki-67 proliferation index of nucleated erythroid cells in the BM and Hb levels in the peripheral blood in transfusion-independent (TI) and transfusion-dependent (TD) MDS patients. A positive trend was seen between the Ki-67 proliferation index and Hb levels, but did not reach statistical significance. (C) Combination of the determination of the Ki-67 proliferation index and Hb levels for prediction of transfusion-dependence in MDS patients. As visualized by Kaplan–Meier curve analyses, the Ki-67 proliferation index of nucleated erythroid cells in the BM was complementary to Hb levels as predictive parameter for transfusion-dependence in MDS patients. Furthermore, determination of the Ki-67 proliferation index allowed prediction of transfusion-dependence in MDS patients with mildly reduced Hb levels. Due to the limited number of samples per patient group, testing the proportional hazard assumption was not possible. Significance levels of the log-rank test are shown adjacent to the Kaplan–Meier curves. Hb = hemoglobin; MDS = myelodysplastic syndrome.

Kaplan–Meier curve analyses revealed that MDS patients with a Ki-67 proliferation index of nucleated erythroid cells in the BM equal or lower than 28% or Hb levels in the peripheral blood equal or lower than 9.3 g/dL showed a significantly higher risk of developing transfusion-dependence within 1 year (Suppl. Figure S1; *P* = 0.015 and *P* = 0.013, respectively). Suppl. Table S2 shows that the Ki-67 proliferation index (*P* = 0.016; hazard ratio (HR) = 0.97) and Hb levels (*P* = 0.020; HR = 0.69) at initial diagnosis were independent predictors of transfusion-dependence of MDS patients within 1 year after diagnosis.

The risk for development of transfusion-dependence was highest in MDS patients with simultaneously very low Ki-67 and Hb levels and was similar to that of the Ki-67 very low/Hb mildly reduced patient group (*P* = 0.14) (Figure 1C). Strikingly, MDS patients with mildly reduced Hb levels at diagnosis that displayed a very low Ki-67 proliferation index of nucleated erythroid cells showed a significantly higher risk for development of transfusion-dependence than patients with mildly reduced Ki-67 and Hb levels at diagnosis (*P* = 0.002).

The observed reduction in the proliferative capacity of nucleated erythroid cells in transfusion-dependent MDS patients is in accordance with the observations of Matarraz et al,^10^ who also found a reduction of proliferating nucleated erythroid cells in transfusion-dependent versus transfusion-independent patients by using DNA quantification. These authors also concluded that this reduced proliferative activity of nucleated erythroid cells at diagnosis may result in future transfusion-dependence in MDS patients.^11^ In the underlying study, the Ki-67 proliferation index of nucleated erythroid cells in the BM was identified as a potential parameter for prediction of future transfusion-dependence in MDS patients in addition to Hb levels. This latter parameter reflects the erythrocyte levels in the peripheral blood.^12^ However, the decreased production of erythroid cells may not be directly detectable through Hb levels, as the average lifespan of erythrocytes is 120 days and existing erythrocytes in the peripheral blood are still viable.^13^ The Ki-67 proliferation index provides additional information on the actual biological activity and production of erythroid cells in the BM for these cases.

Although the Hb level at diagnosis is an important predictor of the development of transfusion-dependence in MDS patients,^14^ the Ki-67 proliferation index was shown to be an independent predictor for transfusion-dependence in MDS patients with mildly reduced Hb levels within 1 year postdiagnosis. Identification of these patients allows for more adequate anticipation on this severe disease complication in clinical practice. This may allow the reduction of burdensome clinical visits for MDS patients and the Ki-67 proliferation index may become an additional prognostic parameter in the WHO-classification-based prognostic scoring system and IPSS-R for MDS patients. Validation in multicenter studies must confirm the predictive value of the Ki-67 proliferation index for transfusion-dependence in MDS patients and its future role as a prognostic parameter. The previous optimization of the method for determination of the Ki-67 proliferation index in BM samples of MDS patients allows for straightforward standardization and distribution among other clinical centers. Combining the measurement of Hb levels and the Ki-67 proliferation index with other laboratory or clinical assessments (eg, the Flow Cytometric Scoring System, aberrant marker expression on myeloid blast cells and specific cytogenetic/molecular aberrancies) may further improve the prediction of transfusion-dependence in MDS patients and should thus be investigated in future studies.^15^

## ACKNOWLEDGMENTS

The authors would like to thank the technicians of the subdivision Bone Marrow Diagnostics, Dept. of Clinical Chemistry & Hematology, Zuyderland Medical Center for their valuable assistance. The authors acknowledge the valuable and critical input in discussions regarding statistical analyses by Dr. A. Merry, epidemiologist, Scientific Research Office.

## AUTHOR CONTRIBUTIONS

EMPC, MPGL, NCJW, FSCR, and AHNH designed and conceptualized the study. SGCM and RJMD were responsible for data acquisition. SGCM was responsible for the analysis and interpretation of the data. SGCM wrote the first draft of the letter. All authors contributed to the revision of the letter.

## DISCLOSURES

FCSR is CSO and QA Manager at Nordic-MUbio, Susteren, The Netherlands. All the other authors have no conflicts of interest to disclose.

## Supplementary Material


