# Supplementary material for: The Proliferation Index of Erythroid Cells Predicts the Development of Transfusion-dependence in Myelodysplastic Syndrome Patients With Mildly Reduced Hemoglobin Levels at Initial Diagnosis
Source: Hemasphere. 2022 Nov 9;6(12):e804. doi: 10.1097/HS9.0000000000000804 (PMC9649276; doi:10.1097/HS9.0000000000000804)
Supplement: Supplementary file 1 [file hs9-6-e804-s001.docx]

**Supplementary Figure Legend**

Supplementary Figure S1

Kaplan-Meier curve analyses of the predictive capabilities of the Ki-67 proliferation index and Hb levels for development of transfusion-dependence in MDS patients. Patients were stratified in a Ki-67 very low (≤ 28%), Ki-67 mildly reduced group (> 28%), Hb very low group (≤ 9.3 g/dL) and Hb mildly reduced group (> 9.3 g/dL). A very low Ki-67 proliferation index or very low Hb levels at diagnosis of MDS patients led to a significantly elevated risk for future development of transfusion-dependence.

**Supplementary Figure**

Supplementary Figure S1


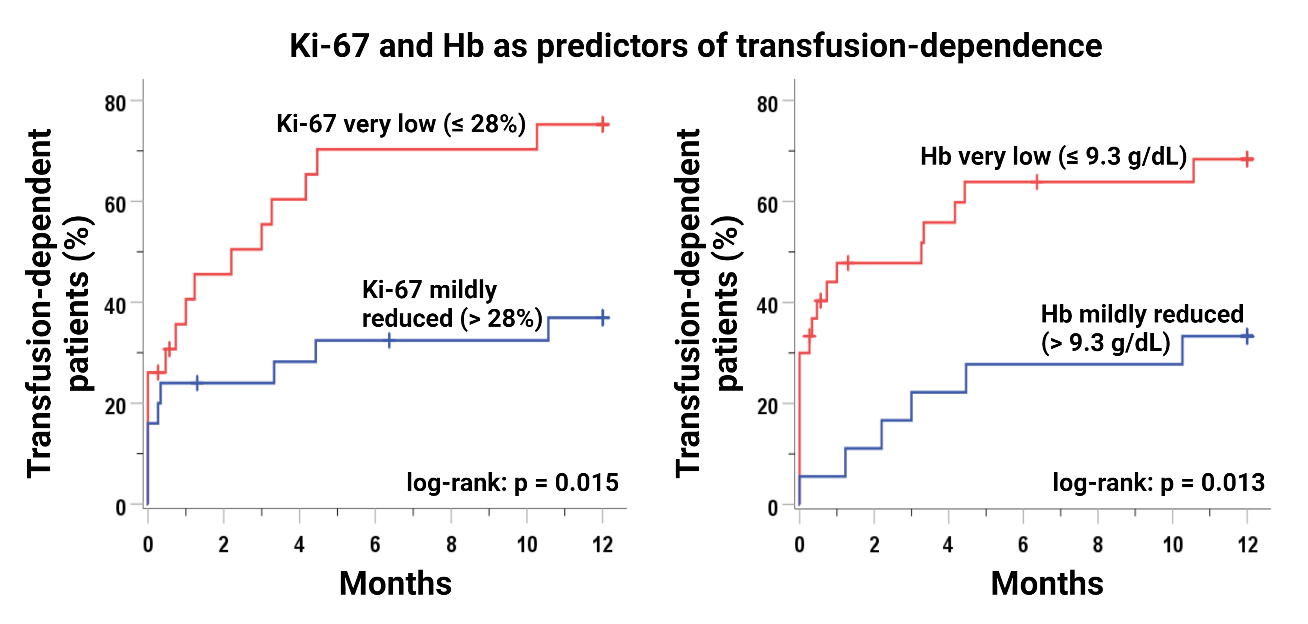


**Supplementary Tables**

Supplementary Table S1: Characteristics of the non-clonal cytopenic control patients and patients with myelodysplastic syndrome (MDS) included in this study.

|  | ***Transfusion independent MDS patients*** | ***n=22*** |  |
| --- | --- | --- | --- |
|  | Age, years (median, range) | 63, 58-93 |  |
|  | Sex (male/female) | 14/8 |  |
|  | **WHO classification** |  |  |
|  | MDS ULD | 1/22 |  |
|  | MDS ULD-RS | 2/22 |  |
|  | MDS MLD | 8/22 |  |
|  | MDS MLD-RS | 5/22 |  |
|  | MDS EB-1 | 4/22 |  |
|  | MDS EB-2 | 2/22 |  |
|  | MDS with 5q deletion | 0/22 |  |
|  | **Clinical presentation** |  |  |
|  | Anemia | 22/22 |  |
|  | Erythropenia | 16/22 |  |
|  | Leukopenia | 6/22 |  |
|  | Thrombocytopenia | 8/22 |  |
|  | Myelodyplasia | 22/22 |  |
|  | Genetic aberrancies | 4/15* |  |
|  | Serum Fe (µmol/L) | 16 (± 12) |  |
|  | Serum Ferritin (µg/L) | 618 (± 466) |  |
|  | Transferrin (g/L) | 2,0 (± 0,4) |  |
|  | Transferrin Saturation (%) | 31 (± 21) |  |
|  | **IPSS-R classification** |  |  |
|  | Very low | 5/15* |  |
|  | Low | 6/15* |  |
|  | Intermediate | 3/15* |  |
|  | High | 0/15* |  |
|  | Very High | 1/15* |  |
|  | ***Transfusion dependent MDS patients*** | ***n=23*** |  |
|  | Age, years (median, range) | 75, 54-86 |  |
|  | Sex (male/female) | 18/5 |  |
|  | **WHO classification** |  |  |
|  | MDS ULD | 0/22 |  |
|  | MDS ULD-RS | 1/22 |  |
|  | MDS MLD | 7/22 |  |
|  | MDS MLD-RS | 2/22 |  |
|  | MDS EB-1 | 6/22 |  |
|  | MDS EB-2 | 6/22 |  |
|  | MDS with 5q deletion | 1/22 |  |
|  | **Clinical presentation** |  |  |
|  | Anemia | 23/23 |  |
|  | Erythropenia | 20/23 |  |
|  | Leukopenia | 14/23 |  |
|  | Thrombocytopenia | 18/23 |  |
|  | Myelodyplasia | 23/23 |  |
|  | Genetic aberrancies | 13/21* |  |
|  | Serum Fe (µmol/L) | 18 (± 12) |  |
|  | Serum Ferritin (µg/L) | 522 (± 328) |  |
|  | Transferrin (g/L) | 1,9 (± 0,3) |  |
|  | Transferrin Saturation (%) | 36 (± 22) |  |
|  | **IPSS-R classification** |  |  |
|  | Very low | 2/21* |  |
|  | Low | 5/21* |  |
|  | Intermediate | 6/21* |  |
|  | High | 3/21* |  |
|  | Very High | 5/21* |  |
| **Genetic aberrancies were not analyzed for 9 MDS cases and as a result IPSS-R classification could not be performed for these cases. Abbreviations: IPSS-R: revised International Prognostic Scoring system; MDS: Myelodysplastic syndrome; MDS-ULD: MDS with unilineage dysplasia; MDS-ULD-RS: MDS with unilineage dysplasia and ringed sideroblasts; MDS-MLD: MDS with multilineage dysplasia; MDS-MLD-RS: MDS with multilineage dysplasia and ringed sideroblasts; MDS-EB1: MDS with excess blasts 1; MDS-EB2: MDS with excess blasts 2.* | | | |

Supplementary Table S2: Univariable and Multivariable Cox-regression shows that the Ki-67 proliferation index and Hb levels at initial diagnosis were independent predictors of transfusion-dependence within 1 year after diagnosis in MDS patients.

|  | **Univariable (p-value)** | **HR (95% CI)** | **Multivariable (p-value)** | **HR (95% CI)** |
| --- | --- | --- | --- | --- |
| **Ki-67+ nucleated erythroid cells (%)** | 0.004 | 0.96 (0.94-0.99) | 0.020 | 0.97 (0.94-0.99) |
| **Age (years)** | 0.184 | 0.97 (0.93-1.0) | 0.959 | 1.00 (0.96-1.10) |
| **Hemoglobin (g/dL)** | 0.0008 | 0.64 (0.46-0.89) | 0.016 | 0.69 (0.51-0.93) |
